# State-Related Changes in MEG Functional Connectivity Reveal the Task-Positive Sensorimotor Network

**DOI:** 10.1371/journal.pone.0048682

**Published:** 2012-10-31

**Authors:** Timothy Bardouille, Shaun Boe

**Affiliations:** 1 Medical Devices Portfolio, National Research Council, Halifax, Nova Scotia, Canada; 2 Department of Diagnostic Imaging, IWK Health Centre, Halifax, Nova Scotia, Canada; 3 Department of Computer Sciences, Dalhousie University, Halifax, Nova Scotia, Canada; 4 School of Physiotherapy, Dalhousie University, Halifax, Nova Scotia, Canada; 5 Department of Psychology, Dalhousie University, Halifax, Nova Scotia, Canada; 6 Heart and Stroke Foundation Centre for Stroke Recovery, Sunnybrook Health Sciences Centre, Toronto, Ontario, Canada; 7 Department of Medicine, Division of Physical Medicine and Rehabilitation, Capital District Health Authority, Halifax, Nova Scotia, Canada; 8 School of Health and Human Performance, Dalhousie University, Halifax, Nova Scotia, Canada; Technical University of Madrid, Italy

## Abstract

Functional connectivity measures applied to magnetoencephalography (MEG) data have the capacity to elucidate neuronal networks. However, the task-related modulation of these measures is essential to identifying the functional relevance of the identified network. In this study, we provide evidence for the efficacy of measuring “state-related” (i.e., task vs. rest) changes in MEG functional connectivity for revealing a sensorimotor network. We investigate changes in functional connectivity, measured as cortico-cortical coherence (CCC), between rest blocks and the performance of a visually directed motor task in a healthy cohort. Task-positive changes in CCC were interpreted in the context of any concomitant modulations in spectral power. Task-related increases in whole-head CCC relative to the resting state were identified between areas established as part of the sensorimotor network as well as frontal eye fields and prefrontal cortices, predominantly in the beta and gamma frequency bands. This study provides evidence for the use of MEG to identify task-specific functionally connected sensorimotor networks in a non-invasive, patient friendly manner.

## Introduction

Coordinated activity of brain regions is essential for integrating multiple information streams into a task-specific strategy for response. Thus, the capacity of the human brain to dynamically involve brain areas in different networks to facilitate neural communication is essential [1], [2]. Consequently, neural networks associated with neurological disorder have been shown to be dysfunctional [3], [4], [5]. Further work is necessary to elucidate the mechanisms that coordinate and control functional and pathological neural networks. Functional connectivity (FC) analysis of non-invasive neuroimaging is essential for extending our knowledge about how neural networks are dynamically modulated.

The most common non-invasive neuroimaging techniques for FC analysis are functional magnetic resonance imaging (fMRI), electro- and magneto-encephalography (EEG/MEG). MEG has several advantages for FC analysis over EEG and fMRI. Whole-head MEG systems provide higher spatial resolution than EEG, as the magnetic fields detected using MEG are less impacted by the varying conductivities of tissues within the head than the electric potentials detected using EEG. In comparison to fMRI, MEG records a direct correlate of neural activity with high temporal resolution, while the blood oxygen level dependent (BOLD) response is a slower, indirect measure of neural activity [6], [7]. A recent study of interest has shown reasonable within-subject correspondence in networks identified using FC measures derived from MEG and fMRI data [8]. The fact that data acquired with MEG reflects the neural activity itself with millisecond resolution provides a means to better understand the underlying mechanisms and directionality of neural communication. Importantly, MEG is a more patient friendly environment with fewer contraindications than MRI, which is critical when considering applications to clinical populations.

The goal underlying FC analysis is to quantify the synchrony between ‘nodes’ in the brain to define a neural network. The underlying assumption is that synchronized activity in spatially distinct neural populations is indicative of neural communication between these areas. Co-activation of both areas by a third neural population is a plausible alternative explanation. In MEG and EEG, synchrony between nodes is often expressed as cortico-cortical coherence (CCC), which measures the consistency over time of the phase difference between two signals as a function of frequency [9], [10]. In fMRI, connectivity is often expressed as the temporal correlation in the BOLD response between nodes [11], [12]. Network connectivity measures have proven effective for revealing a number of neural networks during task performance, as well as in the resting state [13], [14], [15], [16], [17]. Further, network connectivity measured using fMRI is known to modulate between rest and task performance [13]. Of particular importance for the current study, fMRI studies have consistently shown that synchrony between brain areas in the sensorimotor (SM) network is increased during motor tasks, when compared to rest [18], [19]. This task-positive or –negative modulation of connectivity is an important step forward in FC neuroimaging that provides crucial evidence for the functional role of a specific network.

While it is well established that MEG-related FC analysis can identify synchrony during a single task state [8], [9], [16], [20], [21], [22], the task-related modulation of MEG FC is essential to identifying the functional relevance of the identified network. In MEG and EEG, measures such as CCC can be compared between conditions to reveal task- or stimulus-related changes in synchrony, which highlights the nodes in a neural network that have the greatest functional relevance [23], [24]. This solution has been used to study conditionally relevant changes in brain connectivity on time scales on the order of one second. Importantly, this approach can also be applied to compare FC between task states (i.e., *state-related*), wherein the “condition” is the task as a whole, enabling comparative FC analysis to be performed over the course of minutes. For example, reliable changes in FC between rest states (i.e., eyes open versus closed) have been shown using mutual information as a measure of neuronal communication [25]. The current study investigates comparative synchrony between rest and the performance of a motor task to reveal a functionally connected task-positive SM network.

Given the emerging importance of task modulation in elucidating patterns of network connectivity, the purpose of the present study was to provide further validation of this state-related approach to imaging functionally relevant neural networks. To this end, a group of healthy participants underwent MEG scans while at rest and during the performance of a visually cued bilateral motor task. This experimental design provided MEG data during two states, for which existing resting state fMRI literature would predict an increase in synchrony for node-pairs in the SM network during the task state. We compared CCC of anatomically prescribed node-pairs between rest and the performance of a motor task. We hypothesized that the pattern of CCC across the whole-brain would be significantly different between the task state and rest state. Further, we hypothesized that this change would manifest as increased CCC during task performance in functionally relevant nodes of the SM network – specifically, bilateral primary motor and somatosensory cortices (SI/MI), pre-motor cortices (PMC), supplementary motor area (SMA), thalamus and cerebellum (CB). This result would verify the efficacy of the FC approach, which enables the non-invasive, patient friendly investigation of the dynamic role of neural networks in brain function.

## Materials and Methods

### Subjects and Task Paradigm

Eight healthy right-handed adults (4 female, age: 25±4 years) participated in the study. Handedness was quantified based on the Edinburgh Handedness Inventory [26]. Participants provided written informed consent before being enrolled. The study received approval from the National Research Council, Capital District Health Authority and IWK Health Centre research ethics boards. All participants underwent both MEG functional imaging and a T1-weighted MRI for anatomical co-registration.

Eight study blocks were completed with each participant. The first and last blocks were “rest blocks” that provided maximal contrast to the performance of the motor task. In the rest blocks, participants were asked to relax with their eyes closed. Resting block duration was 6 minutes. The second and sixth blocks (“test blocks”) required the participant to perform a visually cued bilateral gripping task that involved controlling the movement of a cursor towards a target (described below). In the third, fourth and fifth blocks, participants practiced the bilateral gripping task at a self-paced rate (“training blocks”). The seventh block involved repetition of the test block with a different target location (“flip block”). Total duration of the motor task blocks varied across participants owing to variability in response time. On average, motor task block duration was approximately 7 minutes. Total time for the MEG session was approximately 1 hour. Only the “rest” and “test” blocks (four blocks in total) were utilized for the purposes of the present paper.

During the test blocks, participants performed a visually cued bilateral gripping task with visual feedback. The goal of the visuomotor task was to accurately move a cursor towards a target located a fixed distance (18.5 ‘virtual cm’) and angle (22° above horizontal) from the initial cursor position. Subjects held two rubber bulbs (inflation bulbs from a sphygmomanometer), one each in the left and right hand, which controlled vertical and horizontal movement of the cursor, respectively. Cursor movement was achieved by gripping the bulbs. The initial change in air pressure generated when a bulb was squeezed was transmitted through pneumatic tubing to a corresponding pressure sensor (model ASCX15DN; Honeywell Sensing and Control, Golden Valley, MN) that converted air pressure to an output voltage. The output voltages corresponding to the left and right hand were translated into horizontal and vertical components of the cursor movement.

The applied pressure was determined from a 300 ms synchronous sampling epoch of the output voltage from the left and right bulb (1000 Hz sampling frequency, USB 6251 M-Series Multi-function DAQ; National Instruments, Austin, TX), 100 ms after the onset of the gripping action. Consequently, the cursor moved along the prescribed trajectory, and the participant received visual feedback regarding the proximity of the cursor to the target (“Accurate”, “Close”, or “Inaccurate”). A 3–4 second rest period (no visual stimulus) occurred before the start of each trial. Participants performed 50 trials of the task during the test blocks.

### MEG Data Acquisition

Before the session, head position indicator (HPI) coils were placed on the participant's forehead (x2) and in front of the ears. The positions of the coils, three anatomical landmarks (nasion and left/right pre-auricular points), and a 150-point head shape were digitized using a Polhemus digitization device (Polhemus Incorporated, Vermont, USA). Self-adhering Ag/AgCl electrodes (EasyCap GmbH, Herrsching, Germany) were placed on the anterior aspect of the participant's left and right forearm in a bi-polar configuration (inter-electrode distance of 2 cm) to monitor muscle activity. The electrodes were positioned to record electromyography (EMG) of the long flexors of the digits (e.g., flexor digitorum superficialis and flexor pollicis longus) during task performance.

MEG, EMG and pressure bulb output voltages were collected during the six task and two rest blocks while the subject was seated in the MEG scanner. The MEG system acquired data from a single magnetometer and two orthogonal planar gradiometers at 102 locations covering the whole head (Elekta Neuromag Oy, Helsinki, Finland). All data were acquired continuously on the same electronics at a sampling rate of 1500 Hz with an inline low-pass filter at 500 Hz for MEG, EMG, and pressure bulb output voltage. Head position indicator coils were activated during all blocks to continuously monitor head movement.

### MRI Processing and Region of Interest Selection

Whole-head anatomical images were acquired using 3D T1-weighted scanning sequences on either a 1.5T (GE Medical Systems, Waukesha, WI) or 4T (Varian Inc., Palo Alto, USA) MRI scanner, based on availability. Co-registration of MEG and MRI coordinate frames was achieved by identifying the nasion and left/right pre-auricular points on the MRI image using the MRILab graphical user interface (Elekta Neuromag Oy, Helsinki, Finland). The head shape digitization supplied both constraint for, and verification of, the co-registration. Anatomical MRIs were skull-stripped [27] and spatially normalized to define the transform between the MRI and Talairach-Tournoux coordinates [28] using the FreeSurfer software suite (Martinos Center for Biomedical Imaging, Massachusetts, USA). A boundary element model of the brain was also generated for use during MEG source estimation [29].

**Figure 1:**
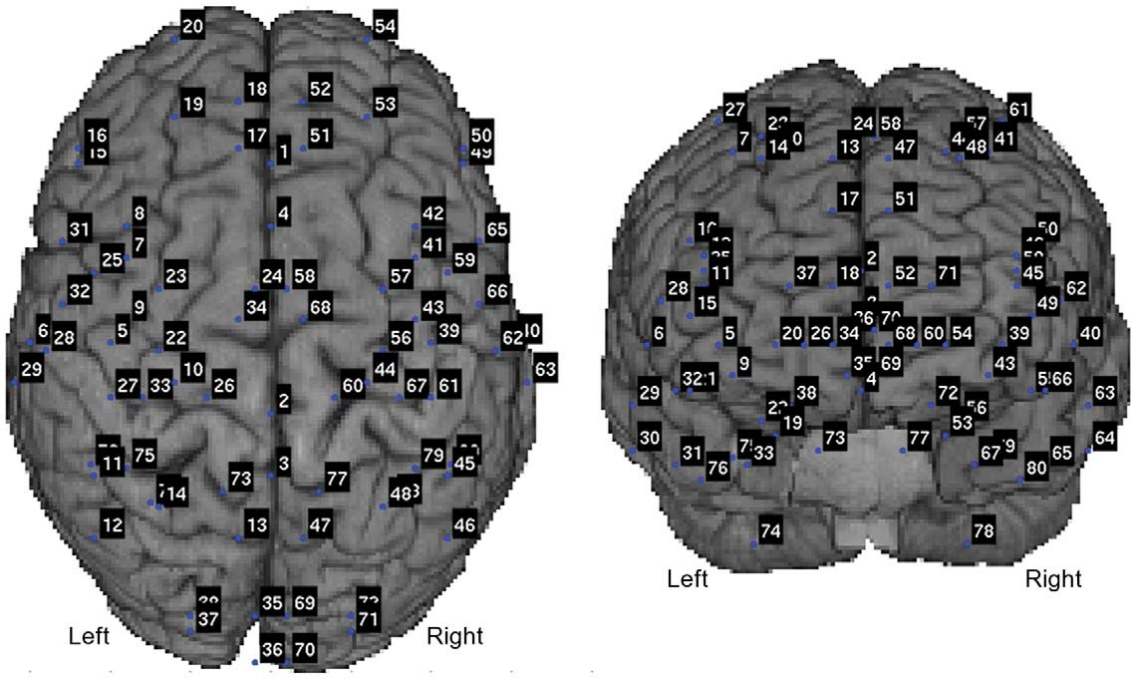
Anatomically prescribed nodes for functional connectivity analysis are shown as blue dots on a template brain for viewing perspective. Numbers associated with cerebellar nodes match the numbers in Table 1.

Seventy-two seed cortical brain areas, as well as eight seed locations in the CB, were selected as nodes to establish a shared coordinate-independent framework for all subjects [30], [31]. Table 1 lists the Talairach-Tournoux coordinates of cerebellar nodes. The coordinates of the other 72 nodes have been previously published [31]. Figure 1 shows the numbered positions of all 80 nodes superimposed on a template brain. For each subject, the Talairach-Tournoux coordinates of these 80 nodes were converted to the MEG head coordinate system using the transformation matrices defined during MEG-MRI co-registration and MRI spatial normalization. These locations defined the nodes for estimating neuronal activity and completing coherence analysis using MEG. Node-pairs for coherence analysis were defined as all 3160 possible pair-wise combinations of nodes.

**Table 1:** Talairach-Tournoux coordinates of cerebellar nodes.

Node	Name	Hemisphere	X [mm]	Y [mm]	Z [mm]
73	Dentate Nucleus	Left	−12	−52	−24
74	Posterior Lobe	Left	−30	−55	−49
75	Cruseus I	Left	−36	−46	−26
76	Cruseus II	Left	−45	−45	−32
77	Dentate Nucleus	Right	12	−52	−24
78	Posterior Lobe	Right	30	−55	−49
79	Cruseus I	Right	36	−46	−26
80	Cruseus II	Right	45	−45	−32

### MEG Source Estimation

Continuous HPI data were analyzed to ensure that the head position was stable over the duration of each rest and task MEG block. Temporal signal-space separation [32] was used for environmental noise reduction. MEG data were low-pass filtered at 100 Hz and down-sampled to 300 Hz to reduce processing time and data storage use. Dataset lengths were matched by including only the first 360 seconds of each block. Estimated source activity for each node (i.e., virtual electrode data) were estimated based on the MEG vendor-supplied beamformer spatial filter (version 2.1) [33], using a realistic boundary element head model for calculation of the forward solution [34], [35].

### MEG Functional Connectivity Analysis

The virtual electrode data at each node were used to calculate the coherence along all node-pairs for each task and rest block as follows. For each block, complex Fourier transforms of the virtual electrode data were calculated on 1.0 s segments every 0.5 s, after applying a Hanning window to each segment. Each Fourier transform contained 256 frequency bins, and generated an estimate of source amplitude and phase over the data segment. Only 51 frequency bins between 4 and 64 Hz (*a priori* frequency range of interest) were used in further analysis. Magnitude-squared CCC for each node-pair and frequency bin was calculated using the complex Fourier transform data, as described elsewhere [10], [36].

### PLS Statistical Analysis

Cortico-cortical coherence results for all subjects and blocks in the 4–64 Hz frequency range were compiled to generate a 4-D data structure (i.e., 4 blocks x 8 subjects x 51 frequency bins x 3160 node-pairs). Mean-centered partial-least squares (PLS) analysis [37] was applied to elucidate latent variables (LVs) that represented significant conditional (i.e., inter-block) differences evident in the CCC data across the group, including effects related to task state, training and time in the scanner. A permutation test with 512 permutations of the inter-block difference tested for a significant change from the null hypothesis (i.e., CCC in all blocks is the same) [38].

Latent variables that showed significant differences across the group were investigated further to reveal which node-pairs and frequency bins reliably expressed the inter-block difference. For each LV, a bootstrap approach with 512 iterations estimated the reliability of the inter-block difference in CCC at each frequency/node-pair combination across the group as a bootstrap ratio (BSR) [39]. The sign of each BSR indicated whether the conditional difference in coherence was expressed in a positive or negative sense (i.e., greater coherence in one group of blocks or the other). A threshold BSR was determined as the 99.9^th^ percentile of all BSR values for the current LV. For each node-pair, the largest (if any) significant positive or negative BSR was determined in the frequency bands defined by the following separation of cortical rhythms; i.e., theta (4–8 Hz), alpha (8–16 Hz), beta (16–32 Hz), and gamma (32–64 Hz).

For each frequency band and LV, we grouped node-pairs with supra-threshold positive and negative BSRs separately. We calculated the node degree – defined as the number of node-pairs that connect each node to the rest of the network – for each node in the resultant positive and negative networks [40]. Nodes with degree of three or more across frequency bands were tabulated as important nodes in the network.

### Virtual Electrode Spectral Power Analysis

Measures of phase synchrony are sensitive to changes in the signal-to-noise ratio of the signals of interest [41]. As such, we also investigated the power spectra of all virtual electrode data for inter-block differences. The power spectrum for each node and frequency bin was calculated by multiplying the complex Fourier transform of the virtual electrode data by its complex conjugate. Spectral power results for all subjects and blocks in the 4–64 Hz frequency range were compiled to generate a 4-D data structure (i.e., 4 blocks x 8 subjects x 51 frequency bins x 80 nodes). As with the CCC data, mean-centered PLS analysis with 512 permutation and 512 bootstrap iterations [37], [38], [39] was applied to elucidate LVs that represented significant inter-block differences evident in the spectral power across the group, including effects related to task state, training and time in the scanner. Latent variables that showed significant differences across the group were investigated further to reveal on which nodes and frequencies the effect was reliably expressed. A threshold BSR was determined as the 99^th^ percentile of all BSR values for the current LV. The interpretation of all CCC results, in terms of functional connectivity, was completed in the context of concomitant modulations in spectral power identified herein.

## Results

### Inter-Block Changes in Connectivity

Two blocks of MEG data during performance of the task (“test1” and “test2”) and two blocks of resting MEG data (“rest1” and “rest2”) were included in the FC analysis. Inter-block changes in CCC between estimates of brain activity at the 80 anatomically prescribed nodes were tested for statistically significant differences from the null hypothesis (i.e., connectivity is the same across all blocks). The PLS approach revealed one significant LV across the group that differentiated CCC between “task” and “rest” blocks (*p*<0.005). Thus, the first LV reveals that FC is modulated between performance of the motor task and rest in this subject group.

### Spatial patterns of connectivity

We further investigated the spectral and spatial pattern of the task-related change in connectivity. The BSRs for the first LV were calculated to evaluate the reliability of the task-related difference across the group between each node-pair and at each frequency bin. Thus, for the first LV, node-pairs and frequency bins with positive supra-threshold (i.e., 99.9^th^ percentile) BSRs reliably showed “task-positive” increases in connectivity. Task-positive increases in CCC occurred mainly in the beta (16–32 Hz) and gamma (32–64 Hz) frequency bands.

**Figure 2:**
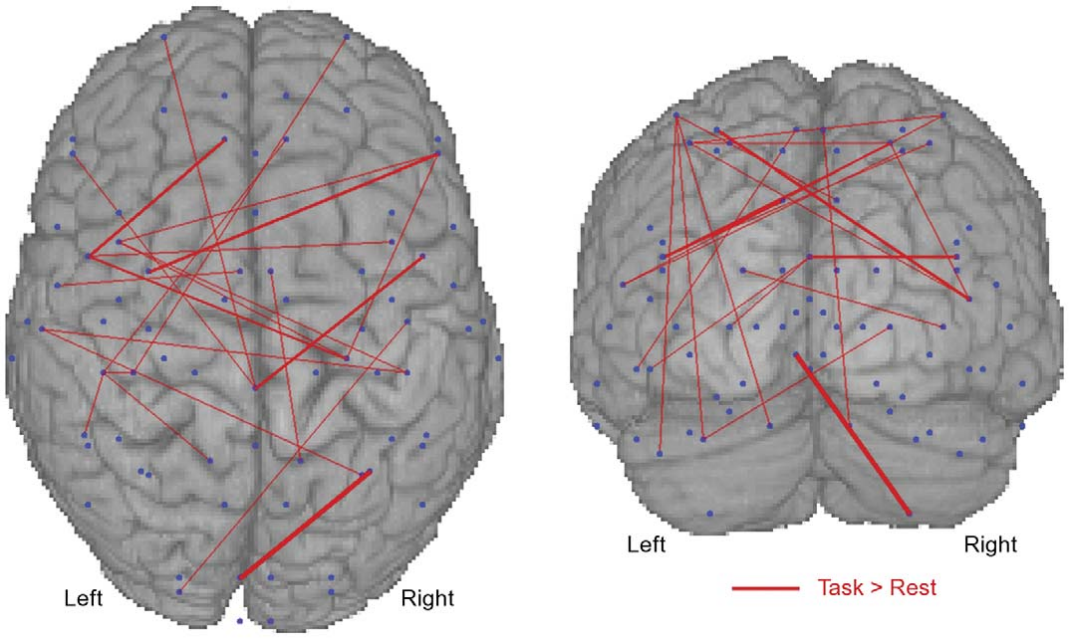
The bootstrap ratios (i.e., reliability; BSR) of task-positive changes in beta (16–32 Hz) band cortico-cortical coherence are shown. Blue dots indicate anatomically prescribed nodes. Red lines indicate coherence was reliably greater during the task. The thickness of the line indicates the magnitude of the BSR. Node-pairs with ratios below the 99.9th percentile are not shown. Data are shown on a template brain for viewing perspective.

**Figure 3:**
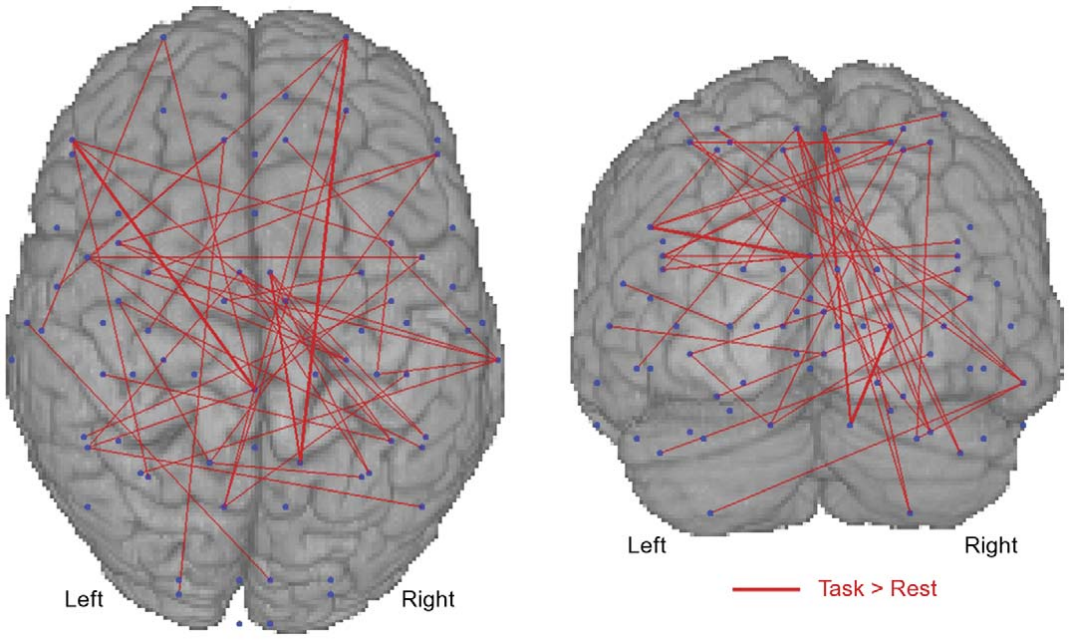
The bootstrap ratios (i.e., reliability; BSR) of task-positive changes in gamma (32–64 Hz) band cortico-cortical coherence are shown. Blue dots indicate anatomically prescribed nodes. Red lines indicate coherence was reliably greater during the task. The thickness of the line indicates the magnitude of the BSR. Node-pairs with ratios below the 99.9th percentile are not shown. Data are shown on a template brain for viewing perspective.

Axial and coronal maps of supra-threshold positive BSR values for the first LV (i.e., task-related increase in synchrony) in the beta (Figure 2) and gamma (Figure 3) bands were generated to reveal the spatial pattern of task-related connectivity. The BSR values are plotted as red lines connecting the relevant nodes to indicate relative increases in coherence during the motor task. The thickness of the line represents the BSR magnitude, with thicker lines indicating greater reliability of the effect across the group. The connectivity maps are superimposed on a 3-D rendered template MRI to provide viewing perspective. Task-positive connectivity in the beta and gamma bands exists most densely at bilateral SI and MI, PMC, SMA, CB, and frontal nodes.

### Task-Positive Functionally Connected Nodes

Nodes of the task-positive network with degree of three or greater (i.e., connections to three or more other nodes) are listed in Table 2. These nodes include bilateral PMC, SI, MI, SMA, and CB, which are all associated with SM processing. As well, the frontal eye fields (FEF) and prefrontal cortices were identified. These areas are involved in eye movement [42] and executive control [43], [44], respectively, which are important processes for the performance of the task utilized in this study. A high degree of task-positive connections to the thalamus (an expected node in the SM network) was not expressed in the data.

**Table 2:** Task-positive network nodes with high degree are listed.

Node	Hemisphere	Degree	Band [Hz]
Supplementary Motor Area	Left	5	32–64
Ventrolateral Premotor Cortex	Left	5	16–64
Posterior Cingulate Cortex	Midline	5	16–64
Middle Temporal Cortex	Right	5	32–64
Dorsolateral Prefrontal Cortex	Left	4	32–64
Primary Somatosensory Cortex	Left	4	16–32
Frontal Polar	Right	4	32–64
Supplementary Motor Area	Right	4	32–64
Dentate Nucleus (Cerebellum)	Left	3	32–64
Frontal Eye Field	Left	3	16–64
Inferior Parietal Cortex	Left	3	32–64
Precuneus	Left	3	32–64
Dorsomedial prefrontal cortex	Left	3	32–64
Cruseus I (Cerebellum)	Right	3	32–64
Primary Motor Cortex	Right	3	16–64
Centrolateral Prefrontal cortex	Right	3	16–64
Ventrolateral Premotor cortex	Right	3	4–8

The name of the underlying anatomical structure, as well as node degree and frequency band is shown.

### Power Spectra

**Figure 4:**
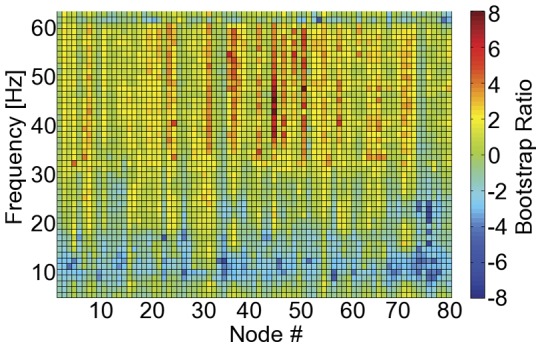
The bootstrap ratios (i.e., reliability; BSR) of task-related changes in spectral power are shown for all nodes (x-axis) and frequency bins (y-axis). A task-related decrease and increase occurs in the alpha (8–16 Hz) and gamma (32–64 Hz) bands, respectively. No reliable task-related change in beta (16–32 Hz) band signal power occurs between rest and performance of the task.

The PLS approach applied to the virtual electrode power spectra identified a single inter-block contrast (LV), which differentiated “test” from “rest” blocks (*p*<0.005). Thus, both spectral power and CCC expressed the same inter-block change. The BSRs for this LV at each node and at each frequency bin – expressing the reliability of the task-related power difference across the group– is shown in Figure 4. Task-related changes in signal power occur predominately in the alpha (decrease) and gamma (increase) bands. Across supra-threshold nodes and frequency bins in the gamma band, the average task-related increase in signal power was 18.7±0.6% (mean ± standard error). The signal power was not reliably different between rest and performance of the task in the beta band (16–32 Hz).

## Discussion

When compared with rest, a task-related increase in FC occurred between brain areas involved in movement and somatosensation (MI/SI), motor planning (PMC/SMA/CB), executive control (PFC) and vision (FEF). Task-positive connectivity manifested as greater CCC mainly in the beta and gamma frequency ranges. The nodes of this task-positive network include many components of the SM network defined based on current resting state [17], [18], [19], [45] and extensive task-related research [16], [46]. The increased coherence between nodes in the SM network during task as compared to rest parallels findings from FC studies performed using fMRI [18], [19]. We did not find any high degree task-positive nodes in the thalamus, although this was expected as MEG reports of thalamic activity are rare, suggesting the technology may be relatively insensitive to this region of the brain [47]. Beyond the SM network, FEF and PFC were also implicated in the task positive network to support the visual aspect of the task, and the integration of feedback into motor planning. Thus, our findings provide evidence for the utility of FC MEG analysis techniques to identify specific task-positive nodes in a functionally connected network.

While functional connectivity maps can be generated based on fMRI data, the neurovascular response is non-specific about the underlying neural mechanisms. Existing electrophysiological literature provides strong evidence for the predominant role of synchrony in beta [48], [49], [50], [51] and gamma [52], [53], [54], [55] cortical oscillations during SM processes. The current work conforms to these previous findings, showing reliable task-related increases in CCC in the beta and gamma frequency bands. Coupling synchrony measures to specific bands of rhythmic neural activity in this way can only be achieved non-invasively with the high temporal resolution available via MEG and EEG. Further, increased beta band FC occurs in the absence of a significant change in signal power relative to the rest block. Demonstrating a task-positive increase in FC in the absence of power change supports the efficacy of this approach to mapping functionally relevant connections within the SM network [41]. The task-related increase in gamma band CCC needs to be considered in the context of the concurrent increase in signal power. Additional work is necessary to develop measures of neuromagnetic synchrony that are stable when concomitant changes in signal power occur.

Combining the mean-centered PLS approach with CCC calculated for MEG beamformer source estimates at anatomically based nodes provided an ideal framework for testing our hypothesis regarding task-related changes in FC. This approach is gaining traction in the FC neuroimaging field for electrophysiological data, and has been applied in an event-related experimental design, requiring the repetitive presentation of a stimulus or performance of a task [23], [24]. In general, FC analysis measures synchrony with respect to an ongoing reference signal such as the activity in a specific brain area, which eliminates the reliance on an event-related design. Examining FC during a single task state has been achieved previously with MEG using CCC and phase locking indices [8], [9], [16], [20], [21], [22]. Further, reliable changes in FC between rest states (i.e., eyes open versus closed) have been shown using mutual information as a measure of neuronal communication [25]. In the current study, the “state-related” approach - measuring changes in synchrony between rest and a specific active task - provides increased specificity in identifying the task-positive neural network. This approach can be readily applied to alternative measures of synchrony such as mutual information or phase locking indices. Thus, the current work provides further validation of a technique for imaging task-specific brain networks that may be applicable to a host of tasks (e.g., navigation, sustained movement, etc.), which have been difficult to access in the past.

One of the challenges in performing FC analysis in MEG (and EEG) is source estimation owing to the reduced spatial resolution of these techniques relative to fMRI. Multiple source estimation techniques, such as the beamformer spatial filter (employed here) and minimum norm estimation, face the challenge that it can be difficult to determine if synchrony between nodes is actually due to two distinct signals being generated in the brain. An alternative explanation is that the source analysis technique has not completely spatially resolved the brain activity at the two distinct locations. In this case, the source estimates contain traces of the same MEG sensor signals – termed “cross-talk” – that can lead to spuriously high levels of coherence between the locations [9]. By looking at relative changes in coherence rather than absolute levels, the FC analysis described here provides an avenue to attenuate “cross-talk”, as well as increase specificity to task-related (i.e., functional) connectivity.

An additional challenge with FC analysis in general is the requirement to calculate the measures over minutes of data. Integrating over time is a necessity in FC analysis given the poor signal-to-noise ratio of the acquired data. This is an inherent weakness of many neuroimaging measures. For example, event-related analysis will average data across numerous stimulus repetitions over the course of minutes. The underlying assumption is that the brain activity is consistent across the measurement interval. Subjects in the current study were actively engaged in the visuomotor task for the majority of the task block. Thus, the increased FC observed during the task block is likely indicative of changes in neural synchrony that support the performance of the task.

The results of this study support the use of MEG-based FC analyses to image task-related changes in neural networks. Further work however is necessary to validate this approach in other experimental paradigms, including those examining cognitive function. In light of several recent studies that show altered patterns of network connectivity in patients with neurological deficits before [4], [5], [56], [57] and after treatment [58], [59], [60], the potential use of MEG as a tool to monitor and direct treatments to facilitate recovery is promising. For instance, establishing relationships between network composition and recovery can aid in guiding treatments designed to restore optimal network connectivity patterns. Additionally, monitoring the changes in network composition as treatment progresses can provide information about treatment efficacy and/or patient responsiveness to treatment. The approach described in the current work may prove useful for monitoring neural networks related to the performance of tasks critical to rehabilitation and recovery.
